# Kaempferol 3-*O*-rhamnoside-7-*O*-rhamnoside is an endogenous flavonol inhibitor of polar auxin transport in *Arabidopsis* shoots

**DOI:** 10.1111/nph.12558

**Published:** 2013-10-25

**Authors:** Ruohe Yin, Kerstin Han, Werner Heller, Andreas Albert, Petre I Dobrev, Eva Zažímalová, Anton R Schäffner

**Affiliations:** 1 Institute of Biochemical Plant Pathology, Helmholtz Zentrum München85764, Neuherberg, Germany; 2 Research Unit Environmental Simulation, Helmholtz Zentrum München85764, Neuherberg, Germany; 3 Institute of Experimental Botany, Academy of Sciences of the Czech RepublicPrague 6, Czech Republic; 4 Present address: Department of Botany and Plant Biology, University of Geneva, Sciences III1211, Geneva 4, Switzerland

**Keywords:** *Arabidopsis thaliana*, flavonol biosynthesis, flavonol glycoside, flavonol glycosyltransferases, plant growth, polar auxin transport

## Abstract

1. Polar auxin transport (PAT) plays key roles in the regulation of plant growth and development. Flavonoids have been implicated in the inhibition of PAT. However, the active flavonoid derivative(s) involved in this process *in vivo* has not yet been identified. Here, we provide evidence that a specific flavonol bis-glycoside is correlated with shorter plant stature and reduced PAT.
2. Specific flavonoid-biosynthetic or flavonoid-glycosylating steps were genetically blocked in *Arabidopsis thaliana*. The differential flavonol patterns established were analyzed by high-performance liquid chromatography (HPLC) and related to altered plant stature. PAT was monitored in stem segments using a radioactive [^3^H]-indole-3-acetic acid tracer.
3. The flavonoid 3-*O*-glucosyltransferase mutant *ugt78d2* exhibited a dwarf stature in addition to its altered flavonol glycoside pattern. This was accompanied by reduced PAT in *ugt78d2* shoots. The *ugt78d2*-dependent growth defects were flavonoid dependent, as they were rescued by genetic blocking of flavonoid biosynthesis. Phenotypic and metabolic analyses of a series of mutants defective at various steps of flavonoid formation narrowed down the potentially active moiety to kaempferol 3-*O*-rhamnoside-7-*O*-rhamnoside. Moreover, the level of this compound was negatively correlated with basipetal auxin transport.
4. These results indicate that kaempferol 3-*O*-rhamnoside-7-*O*-rhamnoside acts as an endogenous PAT inhibitor in *Arabidopsis* shoots.

## Introduction

**Figure 1:**
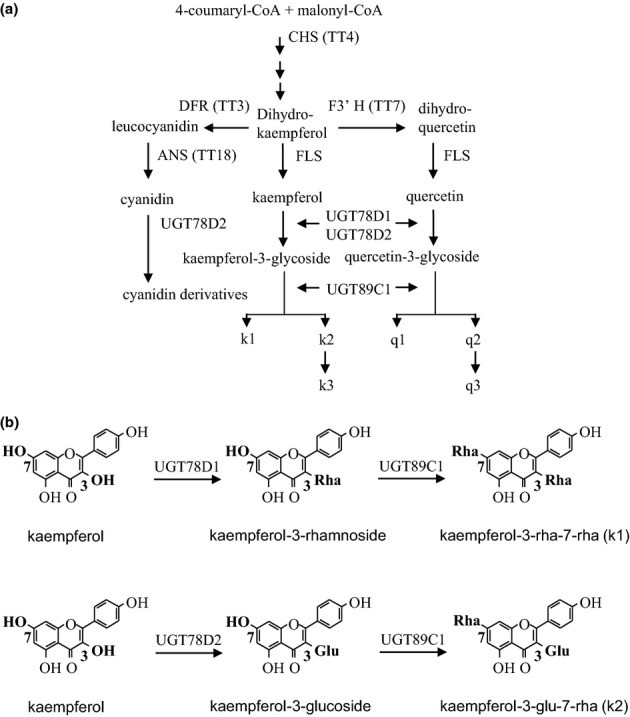
Flavonoid biosynthesis pathway in *Arabidopsis thaliana*. (a) Scheme of flavonoid biosynthesis. CHS (TT4), chalcone synthase; F3′H (TT7), flavonoid 3′-hydroxylase; DFR (TT3), dihydroflavonol 4-reductase; FLS, flavonol synthase; ANS (TT18), anthocyanidin synthase; UGT78D1, flavonol 3-*O*-rhamnosyltransferase; UGT78D2, flavonoid 3-*O*-glucosyltransferase; UGT89C1, flavonol 7-*O*-rhamnosyltransferase. As shown by Yin *et al*. (2012), the combined loss of UGT78D1 and UGT78D2 does not imply an accumulation of flavonol aglycones because of a feedback inhibition of flavonol biosynthesis. (b) Glycosylation reactions catalyzed by UGT78D1, UGT78D2 and UGT89C1 (Jones *et al*., 2003; Tohge *et al*., 2005; Yonekura-Sakakibara *et al*., 2007). Abbreviations: Kaempferol (k); rhamnoside (rha); glucoside (glu). k1, k-3-*O*-rha-7-*O*-rha; k2, k-3-*O*-glu-7-*O*-rha; k3, k-3-*O*-[rha (1->2 glu)]-7-*O*-rha; q1, q2 and q3 are quercetins structurally equivalent to k1, k2 and k3, respectively.

The phytohormone auxin, represented predominantly by indole-3-acetic acid (IAA), plays a crucial role in plant growth and development. Auxin needs to be transported from the sites of synthesis, mainly in the apices and young leaves, to the distal part of the plant to exert its function (Berleth *et al*., 2007). Auxin transporters, including ABCB proteins, AUX1/LAX family members and PIN proteins (Noh *et al*., 2001; Friml, 2003; Petrasek *et al*., 2006; Yang *et al*., 2006; Zažímalová *et al*., 2010; Peer *et al*., 2011), are responsible for the auxin fluxes and patterning in plants (Friml *et al*., 2003). Flavonoids, phenylpropanoic secondary metabolites, have been implicated in the blocking of auxin transport (Peer & Murphy, 2007). The supply of flavonols to detached zucchini hypocotyls resulted in decreased polar auxin transport (PAT) (Jacobs & Rubery, 1988). In *Arabidopsis*, PAT was increased in *transparent testa4* (*tt4*), a flavonoid-deficient mutant defective in the first step of flavonoid production (Shirley *et al*., 1995; Buer & Muday, 2004; Peer *et al*., 2004) (Fig.1). Accordingly, *tt4* roots exhibited delayed gravitropism, which was reversed by chemical complementation by naringenin, an intermediate of flavonoid biosynthesis (Buer & Muday, 2004). By contrast, PAT was reduced in the flavonol over-production mutant *tt3* defective in dihydroflavonol reductase (Fig.1), consistent with an inhibitory role of flavonols in PAT (Peer *et al*., 2004). Despite these and other substantial pieces of evidence supporting a role of flavonols in the modulation of auxin transport (Kuhn *et al*., 2011; Lewis *et al*., 2011; Grunewald *et al*., 2012), neither specific flavonol aglycones nor their conjugates active in this process *in vivo* have been identified so far.

The difficulty in relating specific flavonols to auxin transport modulation is, in part, a result of the complex flavonol modification *in planta*. Flavonol aglycones are intensively modified by UDP-dependent glycosyltransferases (UGTs), which include UGT78D1, UGT78D2, UGT78D3, UGT73C6 and UGT89C1 in the model plant *Arabidopsis thaliana* (Fig.1) (Jones *et al*., 2003; Tohge *et al*., 2005; Yonekura-Sakakibara *et al*., 2007, 2008). The glycosides are distributed in an organ-specific manner. In contrast with the complex flavonol profile in flowers, it is rather simple in inflorescence stems (Yonekura-Sakakibara *et al*., 2008; Stracke *et al*., 2010b). Thus, the inflorescence stem, which is implicated in basipetal auxin movement, is the optimal organ for searching for the flavonol derivative(s) active in auxin transport modulation.

Here, we show that the loss of the flavonoid 3-*O*-glucosyltransferase UGT78D2 resulted in an altered flavonol glycoside pattern and reduced PAT in shoots, which was accompanied by a reduced plant height and increased branching. Blocking of flavonoid biosynthesis and/or glycosylation at specific steps clearly related the enhanced accumulation of kaempferol 3-*O*-rhamnoside-7-*O*-rhamnoside (k1) to the growth defects of *ugt78d2*. Through analyses of auxin transport in several genotypes, which contained different levels of k1, an inverse correlation between basipetal auxin transport and k1 level was identified. Therefore, we propose that k1 acts as an endogenous auxin transport inhibitor in *Arabidopsis* shoots.

## Materials and Methods

### Plant material and growth conditions

The *ugt78d1* (SAIL_568_F08), *ugt78d2* (SALK_049338), *ugt89c1* (SALK_071113), *tt4* (SALK_020583), *tt7* (GK_349F05) and *tt18* (SALK_028793) mutants are based on *Arabidopsis thaliana* (L.) Heynh., accession Columbia (Col-0). These mutant lines have been described in previous studies (Abrahams *et al*., 2003; Alonso *et al*., 2003; Jones *et al*., 2003; Rosso *et al*., 2003; Tohge *et al*., 2005; Yonekura-Sakakibara *et al*., 2007; Stracke *et al*., 2010b). The *tt3-1* (NASC_N84; L*er* background) and *fls1* (Riken_PST16145; Nö background) mutants have different genetic backgrounds (Shirley *et al*., 1995; Ito *et al*., 2005).

*tt4 ugt78d2*, *tt7 ugt78d2, ugt78d1 ugt78d2*, *ugt89c1 ugt78d2*, *tt18 ugt78d2* and *fls1 ugt78d2* double mutants were generated by genetic crossing and confirmed by PCR genotyping. Plants were grown in a sun simulator chamber, which provides a photobiological environment very close to natural global solar radiation (Seckmeyer & Payer, 1993; Thiel *et al*., 1996). Plants were exposed to photosynthetically active radiation (PAR, 400–700 nm) of 200–230 μmol m^−2^ s^−1^ with a 16 h : 8 h, light : dark regime at a temperature of 22°C and a relative humidity of 70%. Similar phenotypes were observed when grown in a glasshouse with higher natural light intensities at temperatures of 20–25°C (European springtime).

A DR5::β-glucuronidase (DR5::GUS) line (Col background; Sabatini *et al*., 1999) was introgressed into *ugt78d2*; plants were grown on half-strength Murashige and Skoog (MS) medium agar plates supplemented with 1.5% sucrose for 9 d and stained for β-glucuronidase expression for 1 h at 37°C (Deruère *et al*., 1999).

### Genetic complementation of *ugt78d2*

A genomic fragment containing the *UGT78D2* gene was amplified from Col-0 genomic DNA using the primers 5′-GGGGACAAGTTTGTACAAAAAAGCAGGCTTTCGGTCCAAAGGATTTCAG-3′ and 5′-GGACCACTTTGTACAAGAAAGCTGGGTAGATTTTCTGAGCCGTGCAT-3′. The PCR product was cloned into vector pDONR221 using GATEWAY™ (Invitrogen, Karlsruhe, Germany) recombination, confirmed by DNA sequencing and further recombined into pBGW (Karimi *et al*., 2002). The resulting binary vector was used to transform *ugt78d2* knockout mutant plants by the floral dip method (Clough & Bent, 1998). Transgenic plants with a single insertion of the transgene were selected by segregation analysis for experiments.

### Analyses of flavonols

For flavonol extraction, the lower 2.5-cm stem segment of 4-wk-old plants was harvested and immediately frozen in liquid nitrogen. The plant material was thoroughly ground in liquid nitrogen using a mortar and pestle, and further homogenized after the addition of 1 ml of methanol per 100 mg of fresh material in a Douncer on ice. The suspension was incubated for 1 h with moderate rotation at 4°C. The extracts were then clarified by centrifugation at 14 000 ***g*** for 10 min with a table-top centrifuge at 4°C. One-third volume of distilled water was added to the supernatant, vortexed and centrifuged as above. The cleared extract was analyzed using a reverse-phase high-performance liquid chromatography (HPLC) system (Gemini C18 Phenyl, 5 μm, 150 × 4.6 mm; Phenomenex, Aschaffenburg, Germany). Solvent A was 1% acetic acid and solvent B consisted of 89% methanol with 1% acetic acid. The elution gradient program followed a linear gradient from 20% solvent B to 100% solvent B (75 min) at room temperature. The quantification of k1 was referred to a kaempferol aglycone authentic standard (Carl Roth, Karlsruhe, Germany).

### Inflorescence stem gravitropic response assay

Arabidopsis plants were grown on commercial soil substrate (Einheitserde; Einheitserde- und Humuswerke, Sinntal-Altengronau, Germany) for 4 wk in a phytochamber (light intensity, 120 μmol m^−2^ s^–1^; 16 h : 8 h, light : dark cycle; relative humidity, 60%; 23°C). Plants were adapted in darkness for 2 h before gravitropic stimulation. Gravitropic stimulation was begun by turning plants by 90° at 23°C in darkness. Except for the 5 min needed for the measurement of the bending angles at each time point, the whole gravitropic response assay was conducted in darkness.

### Examination of parenchyma cell size of inflorescence stems

Seven-week-old inflorescence stems were fixed in ethanol : glycerol : water (36 : 1 : 10, v/v/v), hand sectioned along the longitudinal axis, stained with astral blue and safranin, and examined under a microscope in bright field mode. Cell length was measured using Cell^P Professional Imaging Software (Olympus Europe, Hamburg, Germany).

### PAT assay

The auxin transport assays were performed as described previously with slight modifications (Okada *et al*., 1991). Segments (length, 2.5 cm) from the basal part of the primary inflorescence stem were inserted into 30 μl of 1 × MS medium (pH 5.5) with 10.5 μM [^3^H]-IAA (460 kBq ml^−1^). Both basipetal and acropetal auxin transport were assayed. One end of the stem fragment (apical end for basipetal auxin transport and basal end for acropetal auxin transport) was dipped into the assay mixture. After 6 h of incubation at room temperature in the dark in a closed vial, a 5-mm segment was cut off from the non-submerged end and measured by scintillation counting. For the inhibition assay, 10 μM of *N*-1-naphthylphthalamic acid (NPA) was added. The measurements without NPA treatment were compared between each genotype by a paired *t*-test.

### IAA quantification

Col-0 and *ugt78d2* plants were grown for 28 d before the inflorescence stems were harvested. The whole inflorescence stem was cut into three segments of equal length, namely the apex, middle and basal segment. The extraction and quantification of free IAA were carried out as described previously, with the endogenous IAA determined by isotope dilution followed by liquid chromatography-mass spectrometry (Dobrev *et al*., 2005; Dobrev & Vankova, 2012).

## Results

### Shoot architecture of *ugt78d2* plants is altered

**Figure 2:**
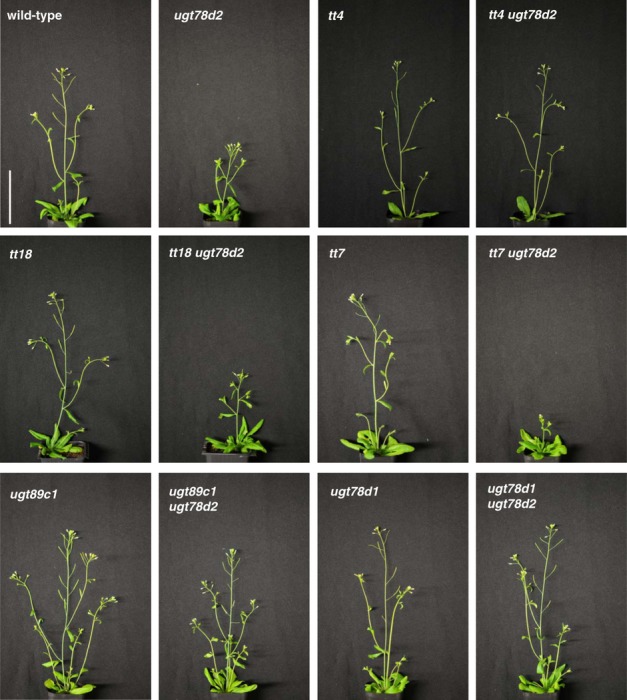
Phenotypes of flavonoid biosynthesis-related mutants. Shoots of *Arabidopsis thaliana* wild-type and the indicated mutants were imaged after 28 d of growth. Bar, 5 cm.

A loss-of-function mutant of the flavonoid 3-*O*-glucosyltransferase UGT78D2 showed a pronounced shoot phenotype (Fig.2). *ugt78d2* mutant plants exhibited a significantly shorter stature soon after bolting (Fig.2). At a later growth stage, the mutants developed more branches with an obvious loss of apical dominance (Supporting Information Table S1). To elucidate the mechanism underlying this dwarfism, the cellular architectures of inflorescence stems were examined. In *ugt78d2*, the parenchymal cell length was reduced significantly when compared with the wild-type. In contrast with stems, other organs were not obviously affected. The lengths of hypocotyls and siliques of *ugt78d2* were similar to those of the wild-type (Table S1). Young seedlings and root systems developed in the same way in both genotypes (Fig. S1) Transformation of *ugt78d2* with a genomic fragment covering the complete *UGT78D2* gene rescued plants from growth disturbances and restored the wild-type phenotype (Fig. S2). Thus, the lesion in *UGT78D2* was responsible for the observed shoot growth defects.

### The *ugt78d2* growth phenotype is flavonol dependent

**Figure 3:**
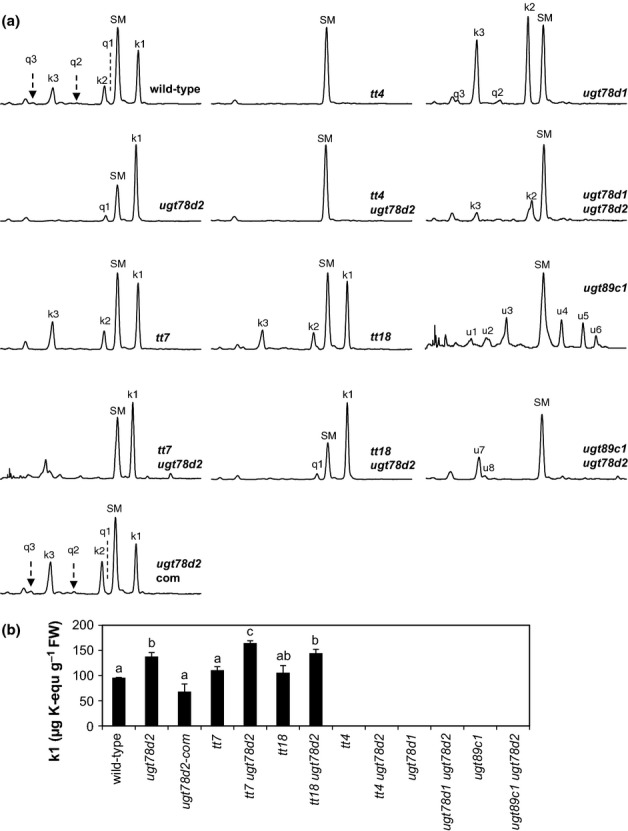
Flavonol glycoside pattern in *Arabidopsis thaliana* mutants affecting flavonoid biosynthesis and conjugation. (a) Flavonols extracted from stems (lower region of 2.5 cm) of the wild-type and flavonoid metabolism-related mutants. The scale of all representative high-performance liquid chromatograms is identical. Peaks u1–u8 represent unknown flavonoids. SM, sinapoyl malate. (b) Quantification of k1 (Fig.1b) in the wild-type and flavonoid metabolism-related mutants. The means of k1 ± SD from four biological samples (a pool of three to five individual stem segments for one sample) are shown. k1 was quantified using kaempferol aglycone as a standard. Letters above the error bars represent a significant difference between genotypes by a paired *t*-test (*P *<* *0.01).

Flavonoids are probable candidates responsible for the *ugt78d2* phenotype, as UGT78D2 has been characterized as a flavonoid 3-*O*-glucosyltransferase and the *ugt78d2* mutant exhibits an altered flavonoid pattern (Lee *et al*., 2005; Tohge *et al*., 2005). Moreover, transformation of *ugt78d2* with the *UGT78D2* gene did not only re-establish the wild-type growth phenotype, but also restored its flavonol pattern (Fig.3a). A flavonoid-deficient *tt4 ugt78d2* double mutant was generated to further check this effect. The growth defects of *ugt78d2* were indeed eliminated in *tt4 ugt78d2*, indicating that they were flavonoid dependent. Moreover, this observation in the flavonoid-deficient background indicated that the growth defects were not related to the repression of specific flavonol-3-*O*-glucoside derivatives (k2, k3, q2 and q3) in *ugt78d2* (Figs2, 3a). In addition to flavonols, UGT78D2 also recognizes anthocyanidins as substrates (Lee *et al*., 2005; Tohge *et al*., 2005). To test the possible involvement of anthocyanins in the development of the *ugt78d2* growth phenotype, an anthocyanin-deficient *tt18 ugt78d2* double mutant was generated. However, loss of the anthocyanidin synthase in the *tt18 ugt78d2* line did not rescue the growth defects of *ugt78d2* (Figs2). The flavonol glycoside pattern of *tt18 ugt78d2* was similar to that of *ugt78d2* (Fig.3a). By contrast, the introgression of *fls1* (accession Nö) into *ugt78d2*, which specifically blocks the biosynthesis of the flavonol moieties, restored wild-type-like growth (Fig. S3). The impact of flavonols was further supported by the slightly repressed growth of the *tt3* mutant in comparison with its genetic background L*er*; the loss of dihydroflavonol reductase in the *tt3* mutant leads to enhanced k1 levels (Fig. S4; Peer *et al*., 2001). Collectively, these observations indicated that the *ugt78d2* growth phenotype was flavonol dependent and that anthocyanins were not responsible for the *ugt78d2* growth defects.

### The *ugt78d2* phenotype is correlated with kaempferol 3-*O*-rhamnoside-7-*O*-rhamnoside

To identify the flavonols responsible for the *ugt78d2* phenotype, a genetic approach using mutants defective in flavonol biosynthetic and/or conjugating steps was employed. In concert with published data (Yonekura-Sakakibara *et al*., 2008; Stracke *et al*., 2010b), six flavonol glycosides were detected in wild-type stems. Three kaempferol glycosides (k1–k3) were the most abundant flavonols, whereas three quercetin glycosides (q1–q3) were present at much lower levels (Fig.3a). No flavonol aglycones were detected in the wild-type and *ugt78d2*, which is in accordance with previous analyses of the single 3-*O*-glycosylation mutants *ugt78d1* and *ugt78d2* (Tohge *et al*., 2007; Yin *et al*., 2012) (Fig.1). Furthermore, even the double mutant *ugt78d1 ugt78d2*, which blocks the major enzymes performing the initial 3-*O*-glycosylation of the flavonol moiety, did not accumulate aglycones (Yin *et al*., 2012). In accordance with the loss of 3-*O*-glucosyltransferase activity in *ugt78d2*, the 3-*O*-glucoside derivatives (k2, k3, q2 and q3) were strongly reduced, whereas the 3-*O*-rhamnoside derivatives (k1 and q1) were elevated, when compared with the wild-type counterpart (Fig.3a). Therefore, both k1 and q1 were positively correlated with the *ugt78d2* growth defects.

A *tt7 ugt78d2* double mutant was generated in order to further distinguish whether the increases in k1 and/or q1 levels were related to the *ugt78d2* growth phenotype. The conversion of kaempferol to quercetin is blocked in *tt7* (Fig.1). As expected, *tt7 ugt78d2* was devoid of q1 and accompanied by an even larger amount of k1 than in *ugt78d2* (Fig.3b). *tt7 ugt78d2* was more severely dwarfed than *ugt78d2*, indicating that a further enhanced k1 level led to a more pronounced phenotype (Fig.2). These observations suggested that the development of the *ugt78d2* growth defects was independent of quercetin derivatives, and that k1 was sufficient to induce the *ugt78d2* phenotype.

k1 contains 3-*O*-rhamnosyl and 7-*O*-rhamnosyl residues (Fig.1b). To further substantiate that k1 was responsible for the observed *ugt78d2* phenotype, additional mutants were employed to selectively inhibit the attachment of 3-*O*- or 7-*O*-rhamnosyl residues to the k1 backbone. The attachment of these residues to the k1 backbone is catalyzed by UGT78D1 and UGT89C1, respectively (Jones *et al*., 2003; Yonekura-Sakakibara *et al*., 2007). As expected, k1 could not be detected in *ugt78d2 ugt89c1*, but unknown flavonol derivatives accumulated (Fig.3a). Importantly, the *ugt78d2* phenotype reverted to a wild-type stature in this double mutant (Fig.2). Likewise, no *ugt78d2*-like phenotype was observed in *ugt78d1 ugt78d2*, which was also devoid of k1 (Figs2, 3a). All of these observations clearly indicated that k1 was indeed the flavonol metabolite inducing the *ugt78d2* phenotype.

### Basipetal auxin transport is reduced in *ugt78d2* plants

**Figure 4:**
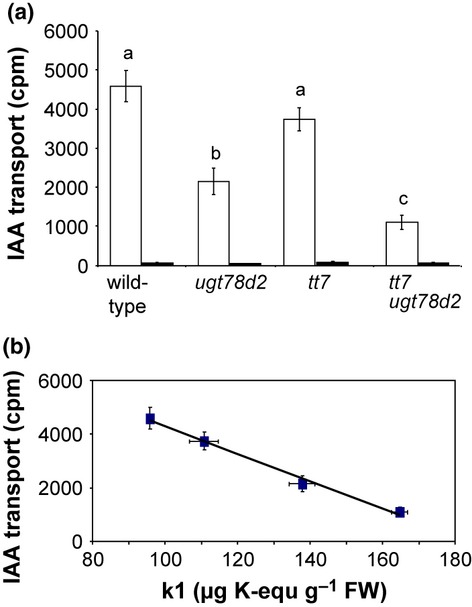
Basipetal auxin transport in inflorescence stems. (a) Basipetal auxin transport in *Arabidopsis thaliana* wild-type, *ugt78d2*, *tt7* and *tt7 ugt78d2* basal stem segments. Data represent radioactivity accumulated in basal segments (means ± SE obtained from 10 individual plants without *N*-1-naphthylphthalamic acid (NPA) treatment and from two individual plants for NPA treatment)). Closed bars, +NPA; open bars, –NPA. Letters above the error bars represent a significant difference between genotypes by a paired *t*-test (*P *<* *0.05). (b) Basipetal auxin transport in relation to k1 levels. Mean value and standard error are plotted. A linear relationship was observed (*R*^2^ = 0.9911).

The dwarf phenotype of *ugt78d2* resembled that of the *abcb1 abcb19* double mutant, which was greatly impaired in auxin transport (Noh *et al*., 2001). As flavonols have been postulated to be endogenous auxin transport inhibitors, we reasoned that the increase in k1 suppressed auxin transport in inflorescence stems, resulting in the growth defects. Therefore, polar transport of [^3^H]-IAA was measured in inflorescence stem segments. NPA-sensitive, basipetal auxin transport in *ugt78d2* was reduced significantly to *c*. 45% of the wild-type level, whereas acropetal auxin transport remained unchanged (Figs4a, S5). Importantly, auxin transport activity further decreased to *c*. 25% of the wild-type level in the *tt7 ugt78d2* double mutant, which accumulated even greater amounts of k1 (Figs3b, 4a). There was a dose-dependent, linear relationship between the k1 level and PAT, when comparing the wild-type, *ugt78d2*, *tt7* and *tt7 ugt78d2* (Fig.4b). The regulation did not affect the transcript level of genes encoding major, putative auxin export proteins, as mRNA levels of *PIN1*, *PIN3*, *PIN7*, *ABCB1* and *ABCB19* were not significantly different in wild-type and *ugt78d2* stems (Fig. S6).

The altered PAT also raised the question of whether there was a general difference in auxin responsiveness. However, several auxin-inducible marker genes were up-regulated by exogenous IAA application in the same manner in *ugt78d2* and the wild-type (Fig. S7).

### Free IAA level is reduced in *ugt78d2* inflorescence stems

To examine whether the reduced auxin transport influenced the concentration of free IAA in *ugt78d2* stems, the free IAA contents of three consecutive stem segments of *ugt78d2* and wild-type plants were compared. The endogenous levels of free IAA in the apical inflorescence segments were not different from those of the wild-type (*P *>* *0.05), whereas the middle and basal stem segments of *ugt78d2* contained lower steady-state IAA levels than the wild-type counterparts (*P *<* *0.05; Table1). This suggested that the decreased IAA content in the lower parts of the inflorescence stem of *ugt78d2* was caused by the reduced basipetal transport of auxin from sites of its biosynthesis in the apex.

**Table 1:** Free indole-3-acetic acid (IAA) quantification in *Arabidopsis thaliana* inflorescence stem segments

	Apical segment	Middle segment	Basal segment
Wild-type	207 ± 50	253 ± 23	247 ± 27
*ugt78d2*	194 ± 17	118 ± 14*	142 ± 18*

Free IAA was measured in three consecutive stem segments (pmol g^−1^ FW). The mean values ± SE of three independent biological samples (one sample is a pool of four to six individual stem segments) are displayed. Statistical analyses were performed by a paired *t*-test for uneven variance for each part of the stem between the two genotypes (^*^, *P *<* *0.05).

### Gravitropism of the inflorescence stem is delayed in *ugt78d2* plants

**Figure 5:**
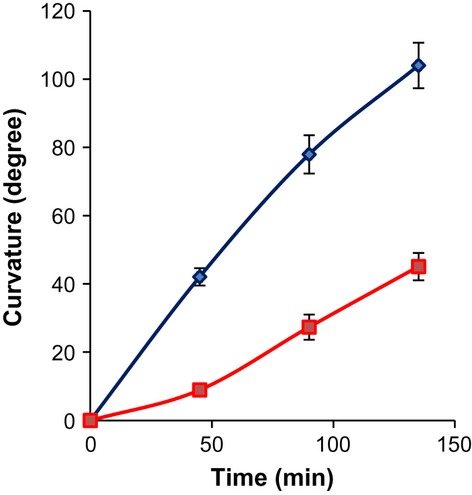
Gravitropic response of wild-type and *ugt78d2* inflorescence stems. Four-week-old *Arabidopsis thaliana* plants were laid down from an upright to a horizontal position in darkness. The curvature of the primary inflorescence stems was measured at different time points to assess the gravitropic response. The angles refer to a horizontal positioning of the tip of the inflorescence (0°) and a fully upright position of the tangential approximation of the bent tip (90°). Wild-type, blue line; *ugt78d2*, red line. Means and SE are shown (*n *=* *15).

The reduction of PAT in the inflorescence stems of *ugt78d2* could also affect tropic responses. Therefore, gravitropism was analyzed in the mutant. Four-week-old plants were laid down horizontally in the dark to exert a gravistimulus, which would lead to an upward bending of the inflorescence stems. The bending angle of the inflorescence stem was monitored at different time points after the gravistimulus. *ugt78d2* exhibited a strongly delayed gravitropic response, indicating that the repressed auxin transport was correlated with a reduced gravitropism in *ugt78d2* inflorescence stems (Fig.5).

## Discussion

Over the past decade, strong evidence, both *in vitro* and *in vivo*, has accumulated implicating flavonols in plant growth regulation and auxin transport modulation (Jacobs & Rubery, 1988; Buer & Muday, 2004; Peer *et al*., 2004; Ringli *et al*., 2008; Santelia *et al*., 2008; Kuhn *et al*., 2011; Lewis *et al*., 2011; Grunewald *et al*., 2012; Buer *et al*., 2013). However, the physiologically active flavonol derivative(s) could not be pinpointed unequivocally. *In vivo* feeding studies were hampered by the possible modification and metabolism of the exogenously added compounds. Similarly, genetic studies affecting either flavonol biosynthesis or flavonol conjugation did not generate effects that could be traced back to a single flavonol moiety (Buer & Muday, 2004; Peer *et al*., 2004; Ringli *et al*., 2008; Buer *et al*., 2013). Nevertheless, the genetic identification of the kaempferol derivative k1 as an endogenous inhibitor of PAT in this work is in agreement with previous studies. These have indicated activity associated with exogenously applied flavonols that could be metabolized to k1 (Jacobs & Rubery, 1988; Mathesius *et al*., 1998). Furthermore, a release from PAT suppression in flavonol-free *tt4* was observed, whereas PAT is further repressed in *tt3*, which exhibits an enhanced flavonol content at the expense of anthocyanins, and, importantly, in *tt7*, which shows specifically increased kaempferol glycoside levels at the expense of quercetins (Buer & Muday, 2004; Peer *et al*., 2004). Furthermore, the formation of k1 is dependent on the 3-*O*-rhamnosyltransferase UGT78D1, which is relatively abundant at the shoot apex in comparison with other tissues and almost absent from roots in agreement with a low k1 level in roots (Jones *et al*., 2003) (Figs S1, S8). Thus, the expression pattern of UGT78D1 correlates with an *in situ* activity of k1 in modulating auxin transport and affecting the shoot phenotype.

Nevertheless, the identification of k1 as an active compound does not exclude an impact of other flavonol derivatives on auxin fluxes. Flavonol biosynthesis is under developmental and organ-specific regulation by MYB and WRKY transcription factors (Stracke *et al*., 2007, 2010b; Grunewald *et al*., 2012). Accordingly, the active compounds could also be dependent on the developmental stage and/or on the organ. WRKY23 has been revealed to be part of a feedback loop of auxin to repress its own transport in roots, as it was induced by auxin and enhanced the biosynthesis of flavonols, in particular of quercetins. Thus, quercetins have been suggested to be active agents in roots (Grunewald *et al*., 2012; Buer *et al*., 2013). This finding was supported by the analysis of the quercetin-less *tt7*, which indicated that no kaempferol derivatives, but rather quercetins, were involved in the suppression of basipetal auxin flux in roots (Lewis *et al*., 2011; Grunewald *et al*., 2012). However, Buer *et al*. (2013) also found a repressed root PAT in both quercetin-accumulating *tt3* and quercetin-deficient *tt7*. Nevertheless, these data and the very low k1 level in roots, as well as the lack of obvious *ugt78d2*-related phenotypes in hypocotyls and roots, including a similar auxin accumulation in mutant and wild-type root tips monitored by a DR5::β-glucuronidase reporter line, strongly support the notion that different flavonol compounds may affect PAT in root and shoot (Figs S1, S9; Table S1).

Suppression of rhamnose biosynthesis in *rol1-2* strongly altered the flavonol profile and induced, for example, hyponastic growth of cotyledons and aberrant leaf cell development. This was related to enhanced auxin accumulation and a repressed auxin (non-IAA) efflux from mesophyll protoplasts (Ringli *et al*., 2008; Kuhn *et al*., 2011). As *rol1-2* growth defects were rescued in *tt4 rol1*, but retained in *tt7 rol1*, they were attributed to kaempferols. However, k1 was not the causal metabolite for this particular phenotype, as *rol1 ugt78d1* lacking k1 retained the *rol1* cotyledon phenotype (Ringli *et al*., 2008).

Several possible mechanisms have been reported on how flavonols might affect auxin transport. Flavonol biosynthetic mutants led to an altered expression, subcellular localization and perhaps modified dynamics in the plasma membrane of some PIN proteins, which could have been caused by a direct or indirect impact of flavonols on these proteins (Peer *et al*., 2004; Santelia *et al*., 2008). At the transcriptional level, there was no difference in mRNA abundance of several *PIN* as well as *ABCB1* and *ABCB19* genes in *ugt78d2* and wild-type plants (Fig. S6). ABCB1 and ABCB19 are ABC transporters which primarily function in the long-distance auxin transport streams and the movement of auxin out of apical tissues (Bandyopadhyay *et al*., 2007). Therefore, these ABCB transporters are potential targets of k1 *in planta*. Indeed, ABCB1/19 proteins are able to bind the quercetin aglycone (Murphy *et al*., 2002), and inhibition of ABCB1 auxin transport activity by the quercetin aglycone has been demonstrated in *Arabidopsis* protoplasts (Geisler *et al*., 2005). These observations are in agreement with previous *in vitro* experiments showing that flavonol aglycones can compete with the synthetic auxin transport inhibitor NPA for binding to isolated microsomal vesicles, although these results may not necessarily reflect an *in vivo* relevance (Jacobs & Rubery, 1988; Murphy *et al*., 2002). Nevertheless, these studies indicate that flavonol derivatives, including k1, could interact directly with and/or inhibit auxin transport proteins.

Despite these *in vitro* studies, a possible involvement of flavonol aglycones in auxin transport inhibition *in vivo* and, in particular, in the *ugt78d2* growth phenotype appears to be unlikely. This notion is based on two lines of evidence: first, to the best of our knowledge, so far only two of numerous studies have detected flavonol aglycones in *Arabidopsis* by either HPLC (Peer *et al*., 2001) or mass spectrometry (Buer *et al*., 2013); however, the latter authors also discussed inconsistencies with respect to the occurrence and absence of aglycones in different *tt* mutants. Although flavonol aglycones exist at least as biosynthetic intermediates, only minute amounts of the hydrophobic molecules might be tolerated in living cells, and therefore free flavonols are not consistently detected; however, this would not preclude a regulatory role (Yin *et al*., 2012). The important second line of evidence stems from loss-of-function mutants affecting the two major UGTs in *Arabidopsis*, UGT78D1 and UGT78D2, which perform the initial 3-*O*-flavonol glycosylation (Fig.1). The lack of either UGT is compensated by the remaining one, and results in a shift of the flavonol glycoside pattern with no aglycones being detected (this work; Jones *et al*., 2003; Yonekura-Sakakibara *et al*., 2008; Yin *et al*., 2012). The *ugt78d1 ugt78d2* double mutant strongly enhances the chance of flavonol aglycone accumulation, yet no free flavonols were detected (Yin *et al*., 2012) and, more importantly, the *ugt78d2*-dependent growth retardation was reversed in the double mutant (Fig.2).

Flavonols have also been shown to exert their function by modulating the interaction between TWD1, a regulatory protein, and ABCB1 transporters, thus suggesting another possible scenario (Bailly *et al*., 2008). Furthermore, it has been reported that PINOID modulates ABCB1-mediated auxin transport through its kinase activity, and that this effect is reversed by direct quercetin binding, that is, the flavonol acts as an endogenous kinase inhibitor to modulate PAT (Henrichs *et al*., 2012). In summary, these mechanistic studies on auxin transport inhibition allow for both different active flavonol moieties and multiple target proteins. The discovery of k1 as an active molecule *in planta* may serve as a valuable tool to obtain further insight into the detailed mechanism of flavonol-dependent auxin transport inhibition.

Apart from the genetic designation of k1, the dose-dependent PAT repression by k1 further supports its identification as an endogenous PAT inhibitor in *Arabidopsis* shoots (Fig. 4). Interestingly, there is also almost the same quantitative relationship in *ugt78d2* relative to the wild-type between the concentrations of k1 (*c*. two-fold higher in the mutant), endogenous auxin (*c*. two-fold lower in the lower inflorescence stem segments of mutant plants) and basipetal auxin transport (*c*. two-fold lower in the basal part of the inflorescence stem of the mutant) (Figs3b, 4a; Table 4). A further increase in k1 in *tt7 ugt78d2* resulted in more severe growth defects. However, the complete lack of k1 (in *tt4*, *ugt78d1*, *ugt89c1*) did not result in a higher stature of the *A. thaliana* plants, suggesting a threshold for k1-dependent growth repression or an independent limitation of growth of these mutants.

In plants, a minor increase in k1 levels would only lead to moderate PAT inhibition, which might not lead to obvious phenotypic changes (as seen in the shoots of *abcb1* and *abcb*19 single mutants; Noh *et al*., 2001). However, under harsh natural growth conditions, including high light, UV-B irradiation, extreme temperatures and drought, k1 as well as other flavonol glycosides could be strongly increased (Hannah *et al*., 2006; Rausher, 2006; Caldwell *et al*., 2007; Korn *et al*., 2008; Götz *et al*., 2010; Stracke *et al*., 2010a). Thus, regulation of flavonol glycoside levels may constitute a means to adjust plant growth and stature by environmental factors and in ecological contexts.

## Supporting Information

**Fig S1**Morphology and flavonol content of young seedlings. **Fig. S2** Genetic complementation of the *ugt78d2*-dependent shoot growth phenotype. **Fig. S3** Growth phenotype and k1 levels of *fls1 ugt78d2*. **Fig. S4** Growth phenotype and k1 levels of *tt3-1* and L*er*. **Fig. S5** Acropetal auxin transport activity in inflorescence stems of wild-type and *ugt78d2*. **Fig. S6** Expression of auxin transporter genes in *ugt78d2* stem segments relative to wild-type. **Fig. S7** Auxin-responsive gene expression in *ugt78d2* and wild-type. **Fig. S8** Analysis of *UGT78D1* gene expression in plant organs. **Fig. S9** Expression of auxin reporter *DR5::GUS* in wild-type and *ugt78d2* roots. **Table S1** Phenotypic analysis of Col-0 and *ugt78d2* plants
