# Supplementary material for: Kaempferol 3-O-rhamnoside-7-O-rhamnoside is an endogenous flavonol inhibitor of polar auxin transport in Arabidopsis shoots
Source: New Phytol. 2013 Oct 25;201(2):466–75. doi: 10.1111/nph.12558 (PMC4260840; doi:10.1111/nph.12558)
Supplement: Supplementary file 1 — Fig S1Morphology and flavonol content of young seedlings. Fig. S2 Genetic complementation of the ugt78d2-dependent shoot growth phenotype. Fig. S3 Growth phenotype and k1 levels of fls1 ugt78d2. Fig. S4 Growth phenotype and k1 levels of tt3-1 and Ler. Fig. S5 Acropetal auxin transport activity in inflorescence stems of wild-type and ugt78d2. Fig. S6 Expression of auxin transporter genes in ugt78d2 stem segments relative to wild-type. Fig. S7 Auxin-responsive gene expression in ugt78d2 and wild-type. Fig. S8 Analysis of UGT78D1 gene expression in plant organs. Fig. S9 Expression of auxin reporter DR5::GUS in wild-type and ugt78d2 roots. Table S1 Phenotypic analysis of Col-0 and ugt78d2 plants [file nph0201-0466-sd1.pdf]

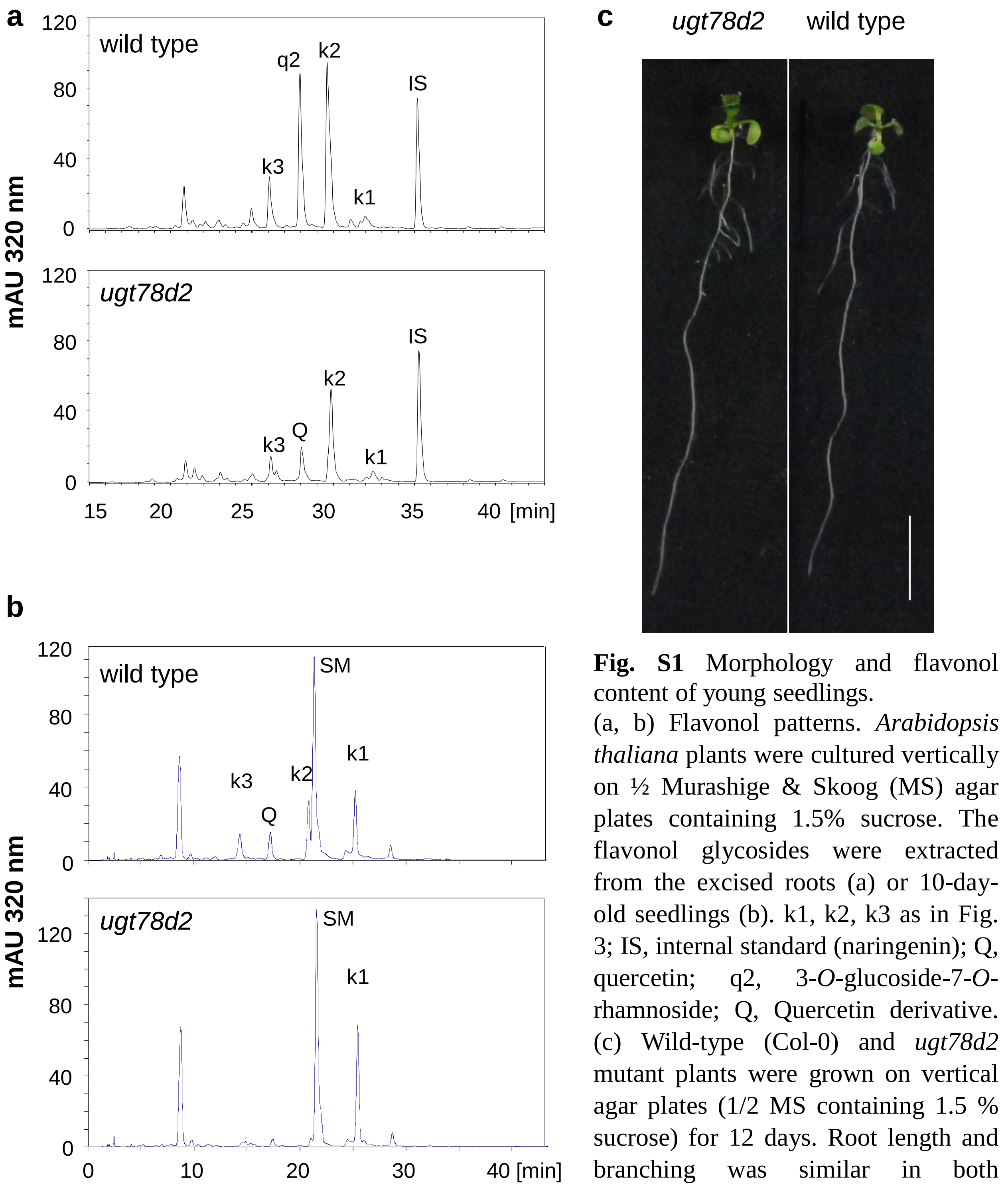

Fig. S1 Morphology and flavonol content of young seedlings.
(a, b) Flavonol patterns. *Arabidopsis thaliana* plants were cultured vertically on ½ Murashige & Skoog (MS) agar plates containing 1.5% sucrose. The flavonol glycosides were extracted from the excised roots (a) or 10-day-old seedlings (b). k1, k2, k3 as in Fig. 3; IS, internal standard (naringenin); Q, quercetin; q2, 3-*O*-glucoside-7-*O*-rhamnoside; Q, Quercetin derivative. (c) Wild-type (Col-0) and *ugt78d2* mutant plants were grown on vertical agar plates (1/2 MS containing 1.5 % sucrose) for 12 days. Root length and branching was similar in both genotypes. Bar, 1 cm.

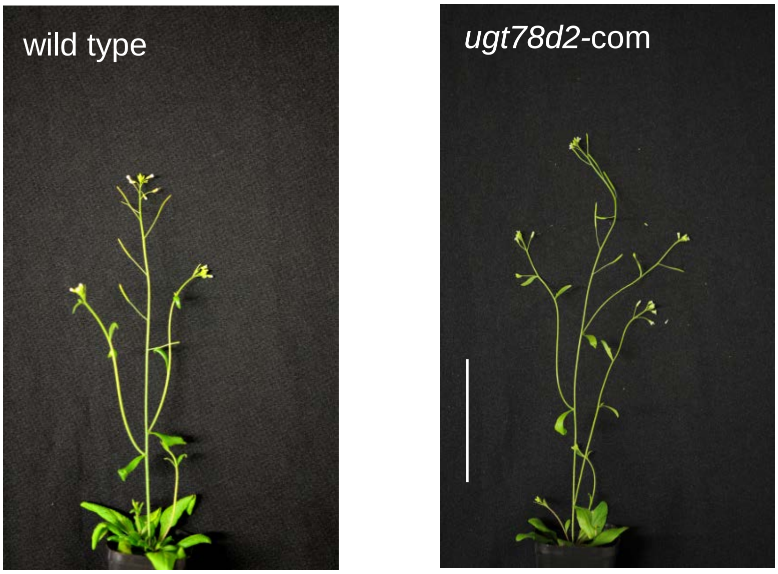

Fig. S2 Genetic complementation of the *ugt78d2*-dependent shoot growth phenotype. *A. thaliana* plants were imaged after 28 days of growth. *ugt78d2*-com indicates a *ugt78d2* mutant genetically complemented with a wild-type *UGT78D2* gene. Scale bar, 5 cm.

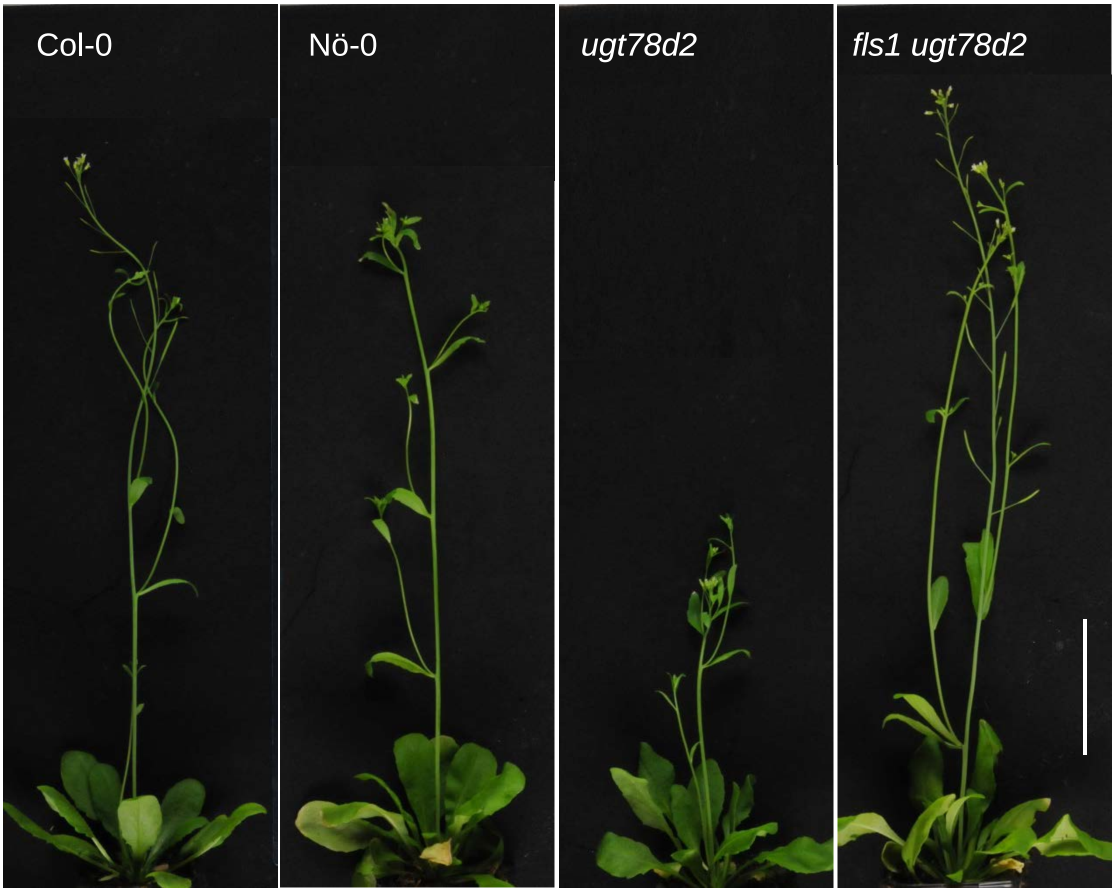

Fig. S3 Growth phenotype and k1 levels of *fls1 ugt78d2*. Blocking the synthesis of the flavonol moiety by introgression of *fls1* reverts the *ugt78d2* growth phenotype. *A. thaliana* accessions Columbia (Col-0) and Nössen (Nö-0) are the corresponding wild-type backgrounds of *ugt78d2* and *fls1*, respectively. Four-week-old plants are shown; bar, 5 cm. The amount of k1 (as in Fig. 3 and S1) was determined by HPLC (n = 5, mean and SD): Col-0, 64.2 ± 8.2; Nö-0, 54.7 ± 11.5; *ugt78d2*, 81.3 ± 15.2; *fls1 ugt78d2*, 4.4 ± 6.9 µg / g fresh weight kaempferol equivalents.

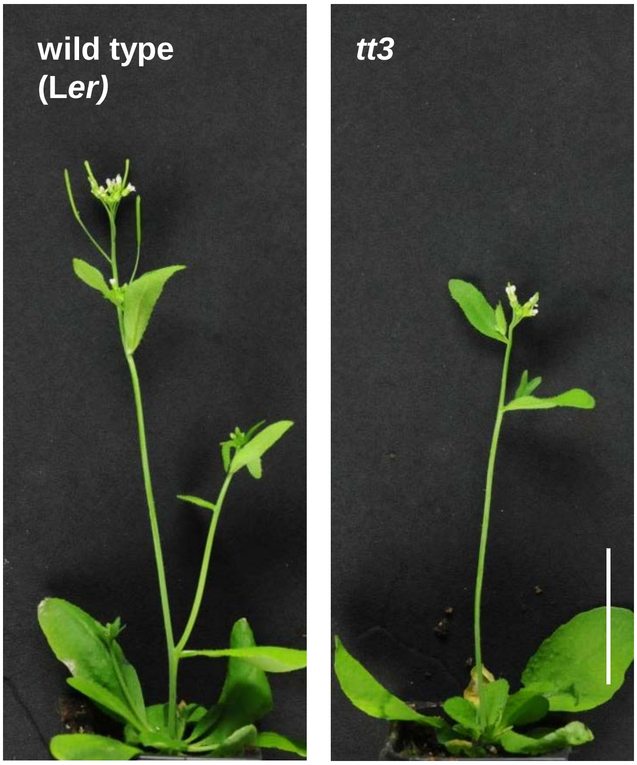

Fig. S4 Growth phenotype and k1 levels of *tt3-1* and L*er*. Four-week-old *A. thaliana* plants are depicted, bar, 5 cm. The amount of k1 in L*er* and *tt3* was determined by HPLC with 16.0 ± 5.7 and 21.6 ± 2.0 µg kaempferol equivalents/ g fresh weight (FW), respectively (SD, n = 5).

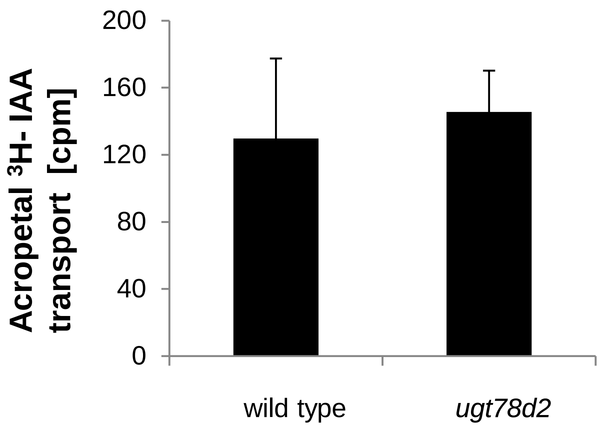

Fig. S5 Acropetal auxin transport activity in inflorescence stems of wild type (Col-0) and *ugt78d2*. Mean values from ten individual plants and standard deviations are shown.

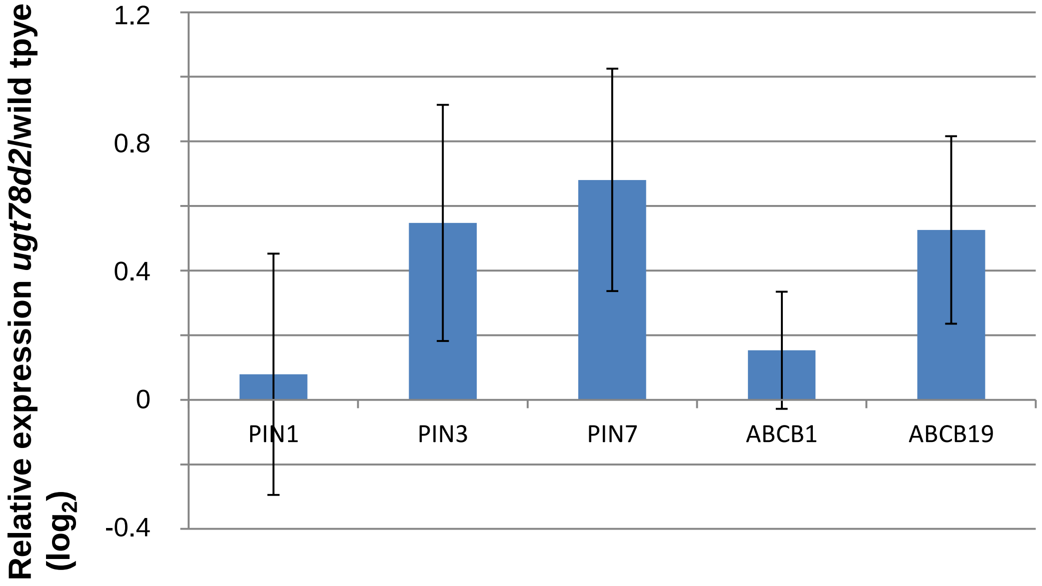

Fig. S6 Expression of auxin transporter genes in *ugt78d2* stem segments relative to wild type. The lower 2.5 cm stem segments were harvested from 4-week-old plants. Expression of the auxin transporter genes *PIN1*, *PIN3*, *PIN7*, *ABCB1* and *ABCB19* was analyzed by RT-qPCR. The data from three independent experiments, each consisting of three independent replicates, were joined and displayed relative to the respective wild-type level (0). None of the genes showed a significant deviation from wild type (P values > 0.05).

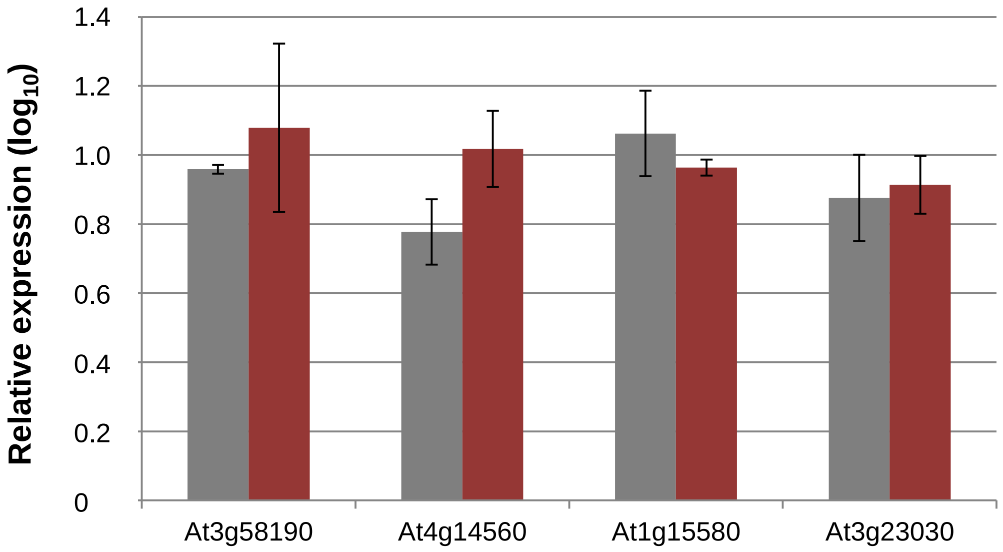

Fig. S7 Auxin-responsive gene expression in *ugt78d2* and wild type. Wild-type and *ugt78d2* seedlings were grown for four days on half-strength MS agar plates. About 30 seedlings per replicate were transferred into 30 ml liquid half-strength MS medium supplemented with 1% sucrose. After three days indole-3-acetic acid (IAA) was added (30 µl 1 mM IAA in 75% ethanol) and incubation continued for 3 h; mock treatments were supplemented with 30 µl 75% ethanol. RNA was isolated and the expression of four known auxin-responsive genes (Genevestigator) was analyzed by RT-qPCR (primers for amplification are given in brackets): At3g58190 (5´-CCCATCTATGGCTGTGTTTCT, 5´-TTGGTGAATCAGCAAAAATCA), At4g14560 (5´-CAACGACTCAACAGAAGAATCTG, 5`-CCGTCCATACTC ACTTTCACAT), At1g15580 (5´-GATACGTTGAAGGAAAGTGAATGT, 5´-CTTCATAATCCTTAACCTCTTGCA), At3g23030 (5´-GGAAACTCGTG ATGAAGAAGAAT, 5´-GTCCATACTCACTTTCACGTAGCT). The induction (auxin treatment *vs*. mock control) is displayed; Col-0, grey bars, *ugt78d2*, red bars; (n = 3, mean values and SD).

Expression signal

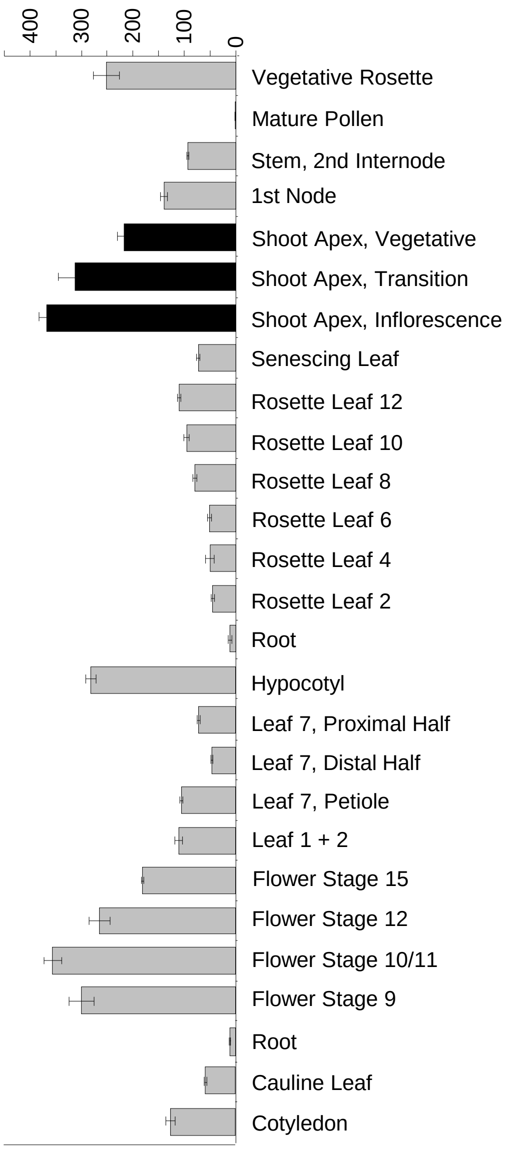

Fig. S8 Analysis of the *UGT78D1* gene expression in plant organs. Public microarray data for *UGT78D1* obtained from *Arabidopsis* eFP Browser (Schmid *et al*., 2005; Winter *et al*., 2007). Particularly high expression was observed in shoot apices (black columns), whereas transcription in roots was very low.

Schmid M *et al.* 2005. A gene expression map of *Arabidopsis thaliana* development. *Nature Genetics* 37: 501-506.

Winter D *et al.* 2007. An "Electronic Fluorescent Pictograph" browser for exploring and analyzing large-scale biological data sets. *PloS One* **2(8)**: e718.

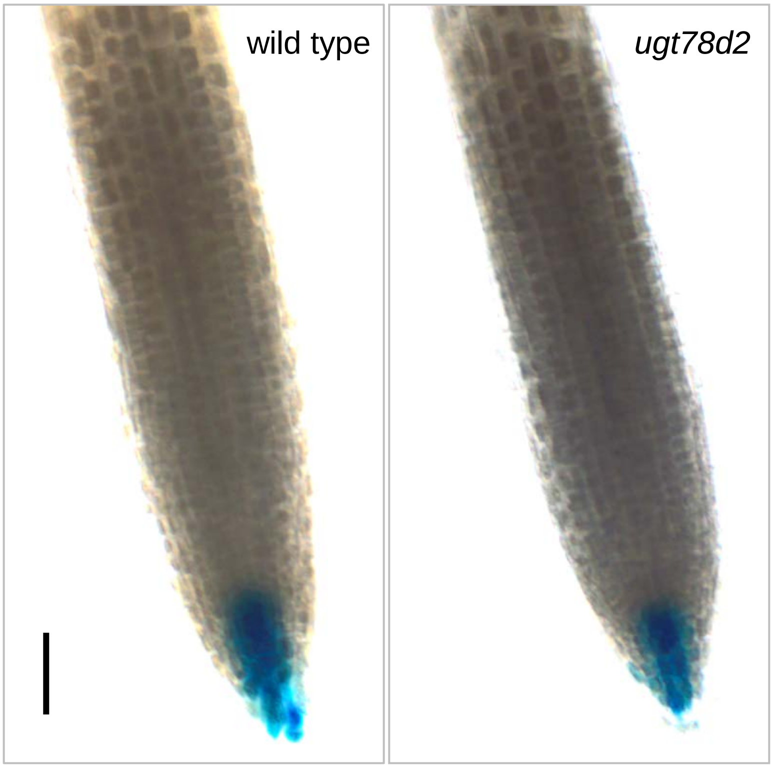

Fig. S9 Expression of auxin reporter *DR5::GUS* in wild-type and *ugt78d2* roots. A $DR5_{pro}$::GUS line in the *A. thaliana* accession Col-0 (wild type) was introgressed into the *ugt78d2* mutant background. Ten-day-old seedlings grown on half-strength MS agar plates were developed for 1 h using 5-bromo-4-chloro-3-indolyl-β-D-glucuronide as a substrate (Deruère *et al*., 1999). Bar, 100 µm.

Deruère J, Jackson K, Garbers C, Soll D, Delong A. 1999. The RCN1-encoded A subunit of protein phosphatase 2A increases phosphatase activity *in vivo*. *Plant Journal* **20**: 389–399.

Table S1 Phenotypic analysis of Col-0 and *ugt78d2* plants.

	**Col-0**	***ugt78d2***
Length of primary inflorescence [cm][1]	35.3 ± 0.9	17.7 ± 0.8 *
Length of lateral branches [cm][1]	22.3 ± 2.4	16.2 ± 0.8 *
Number of lateral branches[1]	5.3 ± 0.4	9.0 ± 0.5 *
Length of siliques [mm][1]	12.2 ± 0.3	12.3 ± 0.3
Length of parenchyma cells in stem [µm][2]	186.1 ± 3.7	158.9 ± 3.3*
Length of hypocotyls [mm][3]	4.7 ± 0.17	5.0 ± 0.16

[1] Seven-week-old *A. thaliana* plants were analyzed (n = 10).

[2] For parenchyma cell length measurement, basal stems from five 33-day-old plants of each genotype were analyzed by microscopy. About 30 cells of each stem segment were measured. The cell length measurement was repeated three times and the results obtained were in accordance with the data shown.

[3] Seedlings were grown on half-strength MS medium supplemented with 1% sucrose and 1% phytagel (Sigma, Germany) for 4 days under weak light (4 µmol m^{-2} s^{-1}) and the length of hypocotyls measured (n = 16).

The mean values ± SE are displayed. Statistical analyses were performed by a paired t-test for uneven variance for each trait between these two genotypes (*P < 0.01).
